# Characteristics of Germline Non-BRCA Mutation Status of High-Risk Breast Cancer Patients in China and Correlation with High-Risk Factors and Multigene Testing Suggestions

**DOI:** 10.3389/fgene.2021.674094

**Published:** 2021-11-30

**Authors:** Yifan Su, Qianlan Yao, Yuyin Xu, Chengli Yu, Jing Zhang, Qian Wang, Jiwei Li, Di Shi, Baohua Yu, Yupeng Zeng, Xiaoli Zhu, Qianming Bai, Xiaoyan Zhou

**Affiliations:** ^1^ Department of Pathology, Fudan University, Shanghai Cancer Center, Shanghai, China; ^2^ Department of Oncology, Fudan University, Shanghai Medical Collage, Shanghai, China; ^3^ Institute of Pathology, Fudan University, Shanghai, China

**Keywords:** breast cancer, high risk, non-BRCA genes, germline mutation, Chinese

## Abstract

**Background:** *Expert consensus on BRCA1/2 genetic testing and clinical application in Chinese breast cancer patients* recommends that *BRCA1/2* testing should be performed in those with clinical risk factors, such as an early onset, triple-negative breast cancer (TNBC) or family history of cancer. With the increasing application of multigene panels, testing for genes beyond *BRCA1/2* has become more prevalent. However, the non-*BRCA* mutation status of Chinese high-risk breast cancer patients has not been fully explored.

**Methods:** A total of 230 high-risk breast cancer patients from Fudan University Shanghai Cancer Center who had undergone peripheral blood germline 72 genes next-generation sequencing (NGS) from June 2018 to June 2020 were enrolled for retrospective analysis. The 72 genes include common hereditary breast cancer genes, such as homologous recombination repair (HRR) genes and other DNA damage repair genes. High-risk factors included: 1) TNBC; 2) male breast cancer; 3) primary bilateral breast cancer; 4) diagnosed with breast cancer at age less than or equal to 40 years; or 5) at least one first- and/or second-degree relative with *BRCA*-related cancer (breast or ovarian or prostate or pancreatic cancer).

**Results:** The germline pathogenic or likely pathogenic mutation rate was 29.6% (68/230) in high-risk breast cancer patients. Among them, 44 (19.1%, 44/230) were identified as harboring *BRCA1/2* mutation, and 28 (12.2%, 28/230) patients carried non-*BRCA* germline variants. Variants were detected in 16 non-*BRCA* genes, including *PALB2* (5, 2.2%), *ATM* (4, 1.7%), *RAD51D* (3, 1.3%), *TP53* (3, 1.3%), *CHEK2* (2, 0.9%), *FANCA* (2, 0.9%) and *ATR*, *BARD1*, *BRIP1*, *ERCC3*, *HOXB13*, *MLH1*, *MRE11*, *PMS2*, *RAD51C*, *RAD54L* (1, 0.4%). Besides, 22 (9.6%, 22/230) patients were non-*BRCA* HRR gene mutation (including *ATM*, *ATR*, *BARD1*, *BRIP1*, *CHEK2*, *FANCA*, *MRE11*, *PALB2*, *RAD51C RAD51D* and *RAD54L*) carriers. Among high-risk factors, family history showed a correlation with both *BRCA* (*p* = 0.005) and non-*BRCA* HRR gene mutation status (*p* = 0.036). In addition, TNBC showed a correlation with *BRCA1* gene mutation status (*p* = 0.038). However, other high-risk factors have not shown significantly related to *BRCA1/2*, non-*BRCA* genes and non-*BRCA* HRR gene mutations (*p* > 0.05). In addition, 312 unique variants of uncertain significance (VUS) were identified among 175 (76.1%, 175/230) patients and 65 different genes.

**Conclusions:** Non-*BRCA* gene mutations are frequently identified in breast cancer patients with high risk factors. Family history showed a correlation with both *BRCA* (*p* = 0.005) and non-*BRCA* HRR gene mutation status (*p* = 0.036), so we strongly suggest that breast cancer patients with a *BRCA*-related family history receive comprehensive gene mutation testing in China, especially HRR genes, which are not only related to high risk of breast cancer, but also potentially related to poly ADP ribose polymerase inhibitor (PARPi) targeted therapy. The exact relationship of rare gene mutations to breast cancer predisposition and the pathogenicity of VUS need to be further investigated.

## Introduction

Breast cancer is considered the most common cancer among females worldwide. According to the epidemiologic analysis report of International Agency for Research on Cancer (IARC), breast cancer had the highest incidence among Chinese female malignant tumors in 2020, with 416,000 patients, accounting for 19.9% of all malignant tumors in women (World Health Organization, 2021). Due to pathogenic or likely pathogenic variants in some tumor suppressor genes, 5–10% of breast cancer cases are hereditary (Carroll et al., 2008), and 15–20% show familial aggregation. Compared to sporadic tumors, hereditary breast cancer is characterized by an early age of onset, male breast cancer, and multiple or bilateral primary tumors (Sung et al., 2017).

Mutations in *BRCA1* and *BRCA2* are closely related to increased susceptibility to breast cancer. Data for the Chinese population showed that the pathogenic or likely pathogenic mutations of *BRCA1/2* were identified in 5.3% unselected Chinese breast cancer patients and in 18.1% familial breast cancer patients (Sun et al., 2017). Besides, the cumulative risk of developing breast cancer by the age of 70 is approximately 37.9% in *BRCA1* mutation carriers and 36.5% in *BRCA2* mutation carriers in China, corresponding to a 10-fold increase compared with the general population (Yao et al., 2016), which indicated that it is necessary to have *BRCA1/2* testing for Chinese breast cancer patients. The Breast Cancer Precision Treatment Committee of the Chinese Medical Doctor Association has issued *expert consensus on BRCA1/2 genetic testing and clinical application in Chinese breast cancer patients* referring to the international guidelines issued by the National Comprehensive Cancer Network (NCCN) and proposed characteristics of breast cancer patients who should be recommended for *BRCA1/2* testing based on clinical risk factors such as age of onset, hormone receptor status and personal or family history of cancer (Wang et al., 2018). For carriers of *BRCA1/2* mutation or their families, there are well-established counseling strategies and management guidelines for early intervention or prevention, such as increased monitoring and consideration of risk-reducing interventions (Kwong et al., 2020). In addition, *BRCA*-related breast cancer is highly sensitive to platinum-based chemotherapy, and these patients can significantly benefit from poly ADP ribose polymerase inhibitor (PARPi) therapy. Nonetheless, more than 60% of breast cancer patients with genetic characteristics or family aggregation do not carry *BRCA1/2* mutations (Telli et al., 2016; Tham et al., 2016; Kwong et al., 2020).

With the application of next-generation sequencing (NGS) over the past 10 years, many non-*BRCA* breast cancer susceptibility genes have been identified in succession, such as *ATM*, *CHEK2*, *PALB2* and *TP53*. Multigene testing is of great significance for breast cancer risk prediction, molecular typing (especially for triple-negative breast cancer) and selection of precise treatment options. A multigene panel enables sequencing of a large number of genes simultaneously. Indeed, more than 200 multigene panels proposed by academic or commercial laboratories have been listed by the NCBI Genetic Test Registry (Thehandbook. Bethes, 2018). Chrystelle Colas et al. summarized the 26 genes most commonly included in the breast cancer multigene panels, including *ATM*, *BARD1*, *BRCA1*, *BRCA2*, *BRIP1*, *CDH1*, *CHEK2*, *GEN1*, *MCPH1, MLH1*, *MRE11A*, *MSH2*, *MSH6*, *NBN*, *NF1*, *PALB2*, *PMS2*, *PTEN*, *RAD50*, *RAD51C*, *RAD51D*, *RINT1*, *SLX4, STK11*, *TP53* and *XRCC2* (Colas et al., 2019). The prevalence and spectrum of germline mutations in breast cancer patients vary across ethnicities. However, only a few studies have reported the prevalence of non-*BRCA* gene mutations in the Chinese population. In addition, the association between clinical risk factors and non-*BRCA* genes in China remains uncertain, and the lack of authoritative guidelines for multigene testing restricts the discovery of more mutation carriers in clinical practice.

Indeed, most of the non-*BRCA* breast cancer susceptibility genes also participate in DNA homologous recombination repair (HRR) pathways, such as *ATM*, *CHEK2* and *PALB2*. HRR is a high-fidelity DNA repair mechanism that is essential for maintaining the integrity of the genome. Evidence showed that pathogenic mutations in non-*BRCA* HRR genes could also help identify susceptibility to familial breast cancer and showed PARPi sensitivity similar to *BRCA1/2* (Mirza et al., 2016; Castroviejo-Bermejo et al., 2018).

In our study, we collected clinicopathological data for 230 high-risk breast cancer patients who had undergone peripheral blood germline gene testing with a 72 multigene panel and retrospectively analyzed the association between risk factors and different gene groups. The aim of this study was to identify the prevalence and spectrum of germline mutations, especially non-*BRCA* and non-*BRCA* HRR genes mutations in high-risk Chinese breast cancer patients, clarify their clinicopathologic characteristics of mutation carriers and provide evidence for proposing the clinical recommendations for genetic testing in high-risk breast cancers.

## Materials and Methods

### Casesand Samples

All cases were collected from Fudan University Shanghai Cancer Center. Data for breast cancer patients who had undergone peripheral blood germline 72 multigene panel testing from June 2018 to June 2020 were collected for statistical analysis. High-risk breast cancer patients were recruited if they fulfilled any one of the following five criteria: 1) triple-negative breast cancer (TNBC); 2) male breast cancer; 3) primary bilateral breast cancer; 4) diagnosed with breast cancer at age less than or equal to 40 years; or 5) at least one first- and/or second-degree relative with *BRCA*-related cancer (breast or ovarian or prostate or pancreatic cancer). All samples were included in the study with approval from the independent ethical committee/institutional review board, and all participants signed informed consent forms. Genomic DNA extracted from peripheral blood were performed using QIAamp DNA blood MidiKit (QIAgen, Valencia, CA) according to manufacturer’s instructions. DNA concentration was measured using Qubit dsDNA assay. Clinicopathological parameters were electronically retrieved from the Hospital Information System (HIS) of Fudan University Shanghai Cancer Center.

### Next-Generation Sequencing Library Preparation and Sequencing

Genome DNA was sheared using Covaris M220, followed by end repairing, phosphorylation and adaptor ligation. DNA fragments were captured using the 72-gene panel (Burning Rock Biotech Ltd.), which covering 370 kb of human genomic regions, and then purified beads (Agencourt AMPure XP Kit, Beckman Coulter, California, United States). Quality and fragment size of such DNA libraries were assessed by Bioanalyzer High Sensitivity DNA Analysis (Agilent). Then libraries were sequenced on Nextseq500 sequencer (Illumina, Inc., California, United States) with pair-end reads. The 72 genes included are *AKT1*, *APC*, *AR*, *ATM*, *ATR*, *BAP1*, *BARD1*, *BRAF*, *BRCA1*, *BRCA2*, *BRIP1*, *CCND1*, *CDK12*, *CDKN1B*, *CDKN2A*, *CDKN2B*, *CHD1*, *CHEK1*, *CHEK2*, *CTNNB1*, *EMSY*, *EPCAM*, *ERCC2*, *ERCC3*, *ERCC4*, *ESR1*, *FAM175A*, *FANCA*, *FANCD2*, *FANCI*, *FANCL*, *FANCM*, *FOXA1*, *GEN1*, *HDAC2*, *HOXB13*, *MLH1*, *MLH3*, *MRE11*, *MSH2*, *MSH6*, *MUTYH*, *MYC*, *NBN*, *NCOR1*, *NCOR2*, *PALB2*, *PIK3CA*, *PIK3CB*, *PIK3R1*, *PMS2*, *POLE*, *PPP2R2A*, *PTEN*, *RAD50*, *RAD51*, *RAD51B*, *RAD51C*, *RAD51D*, *RAD52*, *RAD54L*, *RAF1*, *RB1*, *RNF43*, *RSP O 2*, *SPOP*, *STK11*, *TMPRSS2*, *TP53*, *XRCC2*, *ZBTB16* and *ZNRF3*. Among them, HRR genes include *ATM*, *ATR*, *BARD1*, *BRCA1*, *BRCA2*, *BRIP1*, *CHEK1*, *CHEK2*, *FANCA*, *FANCI*, *MRE11*, *NBN*, *PALB2*, *RAD50*, *RAD51B*, *RAD51C*, *RAD51D* and *RAD54L*.

### Sequence Data Analysis

Sequencing data were aligned to the human genome (hg19) using BWA aligner 0.7.10. Local alignment optimization, variant calling was performed using GATK 3.2, VarScan separately. Variants were filtered using the VarScan fpfilter pipeline, which locations with depth less than 100 were filtered. 5 reads were required for each INDEL alleles, while 8 reads for SNVs. According to allele frequency database (ExAC, 1,000 Genomes, ESP6500 et al.), variants with frequency over 1% were considered as genetic polymorphisms. Variants’ detail information was annotated by ANNOVAR and SnpEff v3.6. DNA translocation analysis was performed using both Tophat2 and Factera 1.4.3.

### Variant Classification and Analysis

Minor allele frequency (MAF) of variant less than 1% was considered for further pathogenicity evaluation. Variants were classified as pathogenic, likely pathogenic, uncertain significance, likely benign and benign according to American College of Medical Genetics (ACMG) guideline (Richards et al., 2015). Pathogenic/likely pathogenic (P/LP) variants were regarded as deleterious mutations with clinical significance. The variants pathogenic determination referred to databases such as the BRCA Exchange database (https://brcaexchange.org/favicon.ico), LOVD database (https://databases.lovd.nl/shared/genes) and ClinVar (http://www.ncbi.nlm.nih.gov/clinvar/) and published papers. Bioinformatic tools including SIFT (http://sift.jcvi.org), Align GVGD (http://agvgd.iarc.fr/agvgd_input.php) and PolyPhen-2 (http://genetics.bwh.harvard.edu/pph2) were used as supplementary evidence to prove that a variant may affect normal function.

### Statistical Analysis

The χ2 test and Fisher’s exact test were employed to evaluate differences in gene mutation frequency across groups as well as the clinicopathological characteristics of mutation carriers. Statistical Product and Service Solutions (SPSS) Statistics 26.0 (IBM institute, Chicago, IL, United States) was used for all statistical analyses. All *p* values were two-sided, and *p* < 0.05 was considered statistically significant.

## Results

### Clinicopathologic Characteristics of Patients

Of 230 breast cancer patients with at least one high-risk factor, the mean age at diagnosis was 37.5 years, with a range from 21 to 79 years; among them 170 (73.9%) had early-onset breast cancer. A total of 103 (44.8%) had a family history of *BRCA*-related cancer (breast or ovarian or prostate or pancreatic cancer) and 21 (9.1%) had a family history of other cancers (such as esophageal, gastric, gallbladder, nasopharyngeal or lung cancer). Two (0.9%) of the breast cancer patients were male. Most breast cancers were invasive ductal carcinoma (209, 90.9%) or ductal carcinoma *in situ* (19, 8.3%); 111 (48.3%) were luminal, 85 (37.0%) were TNBC, and 33 (14.3%) were HER2 positive (HER2+). A total of 5.7% patients were diagnosed with primary bilateral breast cancer. The patients’ clinicopathologic characteristics are summarized in Table 1.

**TABLE 1:** Clinicopathologic Data of 230 High Risk Breast Cancer Patients.

Variable	Multigene patient cohort (N = 230) n (%)	Germline mutation frequency (N = corresponding patients) n (%)			
		Germline mutation carriers	g*BRCA1/2* carriers	Non-*BRCA* germline mutation carriers^a^	Non-*BRCA* HRR germline mutation carriers
**Sex**					
Female	228 (99.1)	68 (29.8)	44 (19.3)	28 (12.3)	22 (9.6)
Male	2 (0.9)	0 (0.0)	0 (0.0)	0 (0.0)	0 (0.0)
**Diagnosis age**					
≤3 years	44 (19.1)	19 (43.2)	14 (31.8)	7 (15.9)	5 (11.4)
31–40 years	126 (54.8)	30 (23.8)	19 (15.1)	12 (9.5)	10 (7.9)
41–50 years	42 (18.3)	15 (35.7)	8 (19.0)	8 (19.0)	6 (14.3)
51–60 years	11 (4.8)	2 (18.2)	1 (9.1)	1 (9.1)	1 (9.1)
>60 years	7 (3.0)	2 (28.6)	2 (28.6)	0 (0.0)	0 (0.0)
**Family history (first or second degree)**					
Breast cancer	96 (41.7)	41 (42.7)	26 (27.1)	17 (17.7)	14 (14.6)
*BRCA* related cancer (other than BC)	7 (3.0)	3 (42.9)	3 (42.9)	1 (14.3)	1 (14.3)
Non-*BRC*A related cancer	21 (9.1)	2 (9.5)	2 (9.5)	1 (4.8)	1 (4.8)
Negative	83 (36.1)	19 (22.9)	11 (13.3)	8 (9.6)	5 (6.0)
Unknown	23 (10.0)	3 (13.0)	2 (8.7)	1 (4.3)	1 (4.3)
**Site of breast tumors**					
Unilateral BC	217 (94.3)	63 (29.0)	39 (18.0)	27 (12.4)	21 (9.7)
Bilateral BC	13 (5.7)	5 (38.5)	5 (38.5)	1 (7.7)	1 (7.7)
**Histology of breast tumors**					
*In situ* carcinoma	19 (8.3)	4 (21.1)	2 (10.5)	2 (10.5)	2 (10.5)
Invasive carcinoma	209 (90.9)	64 (30.6)	42 (20.1)	26 (12.4)	20 (9.6)
Unknown	2 (0.8)	0 (0.0)	0 (0.0)	0 (0.0)	0 (0.0)
**Molecular subtypes of breast tumors**					
Luminal A	8 (3.5)	2 (25.0)	0 (0.0)	2 (25.0)	2 (25.0)
Luminal B (HER2-)	17 (7.4)	6 (35.3)	4 (23.5)	2 (11.8)	1 (5.9)
Luminal B (HER2+)	86 (37.4)	26 (30.2)	15 (17.4)	12 (14.0)	10 (11.6)
HER2+	33 (14.3)	9 (27.3)	6 (18.2)	4 (12.1)	4 (12.1)
TNBC	85 (37.0)	25 (29.4)	19 (22.4)	8 (9.4)	5 (5.9)
Unknown	1 (0.4)	0 (0.0)	0 (0.0)	0 (0.0)	0 (0.0)
**Tumor size**					
≤2 cm	101 (43.9)	29 (28.7)	15 (14.9)	15 (14.9)	13 (12.9)
>2 cm	90 (39.1)	28 (31.1)	21 (23.3)	9 (10.0)	5 (5.6)
Unknown	39 (17.0)	11 (28.2)	8 (20.5)	4 (10.3)	4 (10.3)
**Lymph nodes status**					
Negative	156 (67.8)	50 (32.1)	33 (21.2)	20 (12.8)	17 (10.9)
Positive	57 (24.8)	14 (24.6)	7 (12.3)	8 (14.0)	5 (8.8)
Unknown	17 (7.4)	4 (23.5)	4 (23.5)	0 (0.0)	0 (0.0)
**Metastasis**					
Negative	194 (84.3)	63 (32.5)	40 (20.6)	27 (13.9)	21 (10.8)
Positive	36 (15.7)	5 (13.9)	4 (11.1)	1 (2.8)	1 (2.8)

a 4 patients carried both *BRCA*, and non-*BRCA*, gene germline mutations.

AbbreviationsgBRCA, germline breast cancer susceptibility gene.

### Germline Gene Mutations and Their Distribution in High-Risk Breast Cancer Patients

**FIGURE 1:**
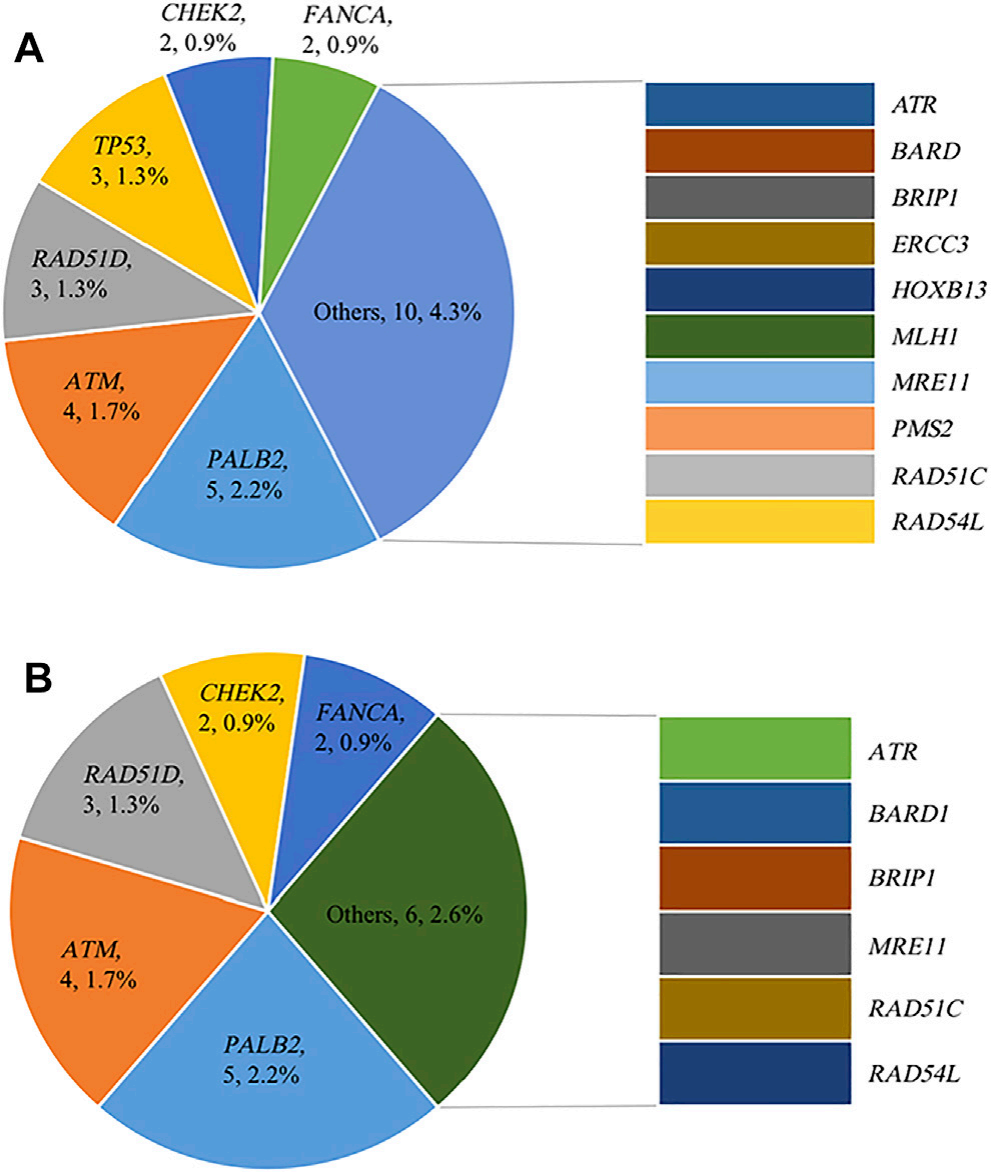
Distribution and frequency of germline mutations **(A)** Distribution and frequency of non-*BRCA* gene germline mutations; **(B)** Distribution and frequency of non-*BRCA* HRR gene germline mutations.

Variants defined as pathogenic or likely pathogenic were selected for analysis. Of 230 high-risk patients, 68 (29.6%, 68/230) were pathogenic or likely pathogenic variant carriers. Table 1 shows the germline mutation frequency of different groups. Forty-four (19.1%, 44/230) high-risk patients were identified as harboring *BRCA* mutations: 33 (14.3%, 33/230) *BRCA1* mutation carriers and 11 (4.8%, 11/230) *BRCA2* mutation carriers. In addition to *BRCA*, 28 (12.2%, 28/230) patients carried non-*BRCA* gene germline variants. In addition, 27.4% (63/230) patients carried HRR gene mutations, of which 22 (9.6%, 22/230) patients were non-*BRCA* HRR gene variant carriers. The distribution and frequency of non-*BRCA* and non-*BRCA* HRR mutant genes are shown in Figure 1. It is worth noting that 5 patients carried more than one pathogenic or likely pathogenic variants simultaneously (Table 2).

**TABLE 2:** Patients Harboring Multiple Variants.

ID	Mutation variant	
N1729354	*BRCA1*:c.2866_2870del; p.Ser956fs	*RAD51D*:c.270_271dup; p.Lys91fs
M2001617	*BRCA1*:c.5470_5477del; p.Ile 1824fs	*RAD51D*:c.556C > T; p.Arg186*
M1921070	*BRCA1*:c.5503C > T; p.Arg 1835*	*MLH1*:c.1038G > C; p.Gln346His
M2002180	*BRCA2*:c.8987del; p.Leu2996fs	*RAD51C*:c.1000G > T; p.Glu334*
M2001784	*MER11*:c.1897C > T; p.Arg633*	*TP53*:c.328dup; p.Arg110fs

### Associations Between High-Risk Factors and Germline Gene Mutation Status

The *BRCA* mutation rate was 19.1% (44/230) in the high-risk groups of breast cancer patients. Among them, 33 (19.4%, 33/170) early-onset patients carried *BRCA* mutation, and 28.2% (29/103) patients with *BRCA*-related family history had *BRCA* mutations; 19 (22.4%) *BRCA* mutation carriers were identified among 85 TNBC patients and 5 (38.5%) carriers among primary bilateral breast cancer patients. However, no mutation was found in the 2 male patients. Family history showed a correlation with *BRCA* mutation (*p* = 0.005), with *BRCA2* mutations having less impact than *BRCA1* mutations. In addition, *BRCA1* mutations showed a correlation with TNBC (*p* = 0.038) (Table 3).

**TABLE 3:** Germline *BRCA1* and *BRCA2* mutation status in patients in different high-risk categories.

			g*BRCA1/2* mutation status						
Number of patients			Non-carriers^a^ (n = 186)	g*BRCA1* carriers (n = 33)	g*BRCA2* carriers (n = 11)	g*BRCA1/2* carriers^b^ (n = 44)			
			**N (%)**	**N (%)**	**N (%)**	**N (%)**	***p*1**	***p*2**	***p*3**
**Early-age onset breast cancer**									
No		60	49 (81.7)	7 (11.7)	4 (6.7)	11 (18.3)	0.533	0.467	0.855
Yes		170	137 (80.6)	26 (15.3)	7 (4.1)	33 (19.4)	—	—	—
**Breast cancer with family of breast/ovarian/prostate/pancreatic cancer**									
No		104	91 (87.5)	9 (8.7)	4 (3.8)	13 (12.5)	0.008	0.226	0.005
Yes		103	74 (71.8)	22 (21.4)	7 (6.8)	29 (28.2)	—	—	—
Unknown		23	21 (91.3)	2 (8.7)	0 (0.0)	2 (8.7)	—	—	—
**Triple-negative breast cancer**									
No		145	120 (82.8)	15 (10.3)	10 (6.9)	25 (17.2)	0.038	0.073	0.341
Yes		85	66 (77.6)	18 (21.2)	1 (1.2)	19 (22.4)	—	—	—
**Male breast cancer**									
No		228	184 (80.7)	33 (14.5)	11 (4.8)	44 (19.3)	0.550	0.730	0.490
Yes		2	2 (100.0)	0 (0.0)	0 (0.0)	0 (0.0)	—	—	—
**Bilateral breast cancer**									
No		217	178 (82.0)	29 (13.4)	10 (4.6)	39 (18.0)	0.069	0.460	0.068
Yes		13	8 (61.5)	4 (30.8)	1 (7.7)	5 (38.5)	—	—	—

Mutations identified as pathogenic or likely pathogenic were enrolled in our analysis. *p*1, non-carriers versus g*BRCA1* carriers; *p*2, non-carriers versus g*BRCA2* carriers; *p*3, non-carriers versus g*BRCA1/2* carriers. Bold values denote two-sided *p* < 0.05.

a Non-carriers included all patients without *BRCA*, mutation.

b *BRCA1/2* carriers included g*BRCA1* carriers and g*BRCA2* carriers.

When considering non-*BRCA* genes, the non-*BRCA* gene and non-*BRCA* HRR gene mutation rates were 12.2% (28/230) and 9.6% (22/230), respectively. Among them, 11.2% (19/170) early-onset patients had non-*BRCA* mutations; 18 (17.5%, 18/103) patients with *BRCA*-related family history were non-*BRCA* mutation carriers; besides, 8 (9.4%, 8/85) TNBC patients and 1 (7.7%, 1/13) primary bilateral breast cancer patients were identified. In addition, 15 (8.8%, 15/170) early-onset patients, 15 (14.6%, 15/103) patients with *BRCA*-related family history, 5 (5.9%, 5/85) TNBC patients and 1 (7.7%, 1/13) primary bilateral breast cancer patients carried non-*BRCA* HRR gene germline mutations. The relevance between non-*BRCA* HRR gene mutations and high-risk factors was lower than that between *BRCA* and high-risk factors. For each high-risk factor, only family history showed a correlation with the non-*BRCA* HRR gene mutation status (*p* = 0.036) (Table 4).

**TABLE 4:** Germline non-*BRCA* mutation status in patients in different categories.

		Germline mutation status			gHRR mutation status		
Number of patients		Non-carriers^a^ (n = 202)	Non-*BRCA* germline mutation carriers (n = 28)		Non-carriers^b^ (n = 208)	Non-*BRCA* gHRR carriers (n = 22)	
		**N (%)**	**N (%)**	***p*1**	**N (%)**	**N (%)**	***p*2**
**Early-age onset breast cancer**							
No	60	51 (85.0)	9 (15.0)	0.436	53 (88.3)	7 (11.7)	0.520
Yes	170	151 (88.8)	19 (11.2)		155 (91.2)	15 (8.8)	
**Breast cancer with family of breast/ovarian/prostate/pancreatic cancer**							
No	104	95 (91.3)	9 (8.7)	0.060	98 (94.2)	6 (5.8)	0.036
Yes	103	85 (82.5)	18 (17.5)	—	88 (85.4)	15 (14.6)	—
Unknown	23	22 (95.7)	1 (4.3)	—	22 (95.7)	1 (4.3)	—
**Triple-negative breast cancer**							
No	145	125 (86.2)	20 (13.8)	0.327	128 (88.3)	17 (11.7)	0.146
Yes	85	77 (90.6)	8 (9.4)	—	80 (94.1)	5 (5.9)	—
**Male breast cancer**							
No	228	200 (87.7)	28 (12.3)	0.597	206 (90.4)	22 (9.6)	0.644
Yes	2	2 (100.0)	0 (0.0)	—	2 (100.0)	0 (0.0)	—
**Bilateral breast cancer**							
No	217	190 (87.6)	27 (12.4)	0.611	196 (90.3)	21 (9.7)	0.813
Yes	13	12 (92.3)	1 (7.7)	—	12 (92.3)	1 (7.7)	—

Mutations identified as pathogenic or likely pathogenic were enrolled in our analysis. *p*1, non-carriers versus non-*BRCA* gHRR, carriers; *p*2, non-carriers versus non-*BRCA*, germline mutation carriers. Bold values denote two-sided *p* < 0.05.; Abbreviations: gHRR, germline homologous recombination susceptibility gene.

a Non-carriers included all patients without non-*BRCA* gHRR, mutation.

b Non-carriers included patients without germline mutation and patients carried *BRCA*, mutations only.

### Non-*BRCA* Gene Mutations

Sixty-eight patients were pathogenic or likely pathogenic mutation carriers. All the pathogenic or likely pathogenic variants were listed in Table 5. Among them, twenty-eight unique non-*BRCA* variants were identified, in which *RAD51D* variant c.270_271dup (p.Lys91fs) occurred twice. As mentioned above, *PALB2* (n = 5), *ATM* (n = 4), *RAD51D* (n = 3) and *TP53* (n = 3) were the top 4 genes among non-*BRCA* genes with the highest mutation rate. In addition, seven variants were identified in non-HRR genes: *ERCC3* c.1854_1867del (p.Glu619fs); *HOXB13* c.179del (p.Pro60fs); *MLH1* c.1038G > C (p.Gln346His); *PMS2* exon14-15cn_del; *TP53* c.328dup (p.Arg110fs); *TP53* c.637C > T (p.Arg213*) and *TP53* c.733G > A (p.Gly245Ser).

**TABLE 5:** List of pathogenic/likely pathogenic variants in 68 patients.

Gene	Mutation variant	Mutation Type	ID	Subtype	Type of cancer	Age of onset	*BRCA*-related family history	Read Depth	Allele Depth	Allele Frequency
*ATM*	c.1402_1403del; p.Lys468Glufs	frameshift	M1904071	TNBC	breast	54	—	372	182	0.52
	c.3475del; p.Ala1159fs	frameshift	N1828231	Luminal A	breast	33	breast	496	223	0.45
	c.6976-1G > C	splice site	M1923378	HER2+	breast	38	breast	607	285	0.47
	exon17-59cn_del	large genomic rearrangement	M1914872	Luminal B (HER2+)	breast	48	breast	—	—	0.99
*ATR*	c.6279_6280del; p.Trp 2094fs	frameshift	M1914650	TNBC	breast	38	—	664	332	0.50
*BARD1*	exon1cn_del	large genomic rearrangement	M1918326	Luminal A	breast	30	breast	—	—	0.59
*BRCA1*	c.1016del; p.Lys339fs	frameshift	M1915861	TNBC	breast	32	breast; ovary	364	197	0.54
	c.1319T > A; p.Leu440^a^	stop gained	M2005589	TNBC	breast	27	—	291	151	0.52
	c.2110_2111del; p.Asn704fs	frameshift	M1917376	Luminal B (HER2+)	bilateral breast	37	breast	317	174	0.55
	c.2481del; p.Gly828fs	frameshift	M1908555	Luminal B (HER2-)	breast	34	breast	258	129	0.50
	c.2491dup; p.Tyr831Leufs	frameshift	M1900311	TNBC	breast	40	breast	3,698	1,664	0.45
	c.2866_2870del; p.Ser956fs	frameshift	N1729354	HER2+	breast	30	breast	496	238	0.48
	c.3689T > G; p.Leu1230^a^	stop gained	M1913565	TNBC	breast	40	-	351	183	0.52
	c.4041_4042del; p.Gly1348fs	frameshift	M2001543	Luminal B (HER2+)	breast	36	breast	384	173	0.45
	c.40dup; p.Val14fs	frameshift	M2005311	TNBC	bilateral breast	29	—	642	321	0.50
	c.4165_4166dup; p. Ser1389Argfs	frameshift	M1819548	TNBC	breast	31	—	2,141	921	0.43^a^
	c.4327C > T; p.Arg1443^a^	stop gained	D2000700	HER2+	bilateral breast	55	breast	622	311	0.50
	c.4357+1G > A	splice site	M1701555	TNBC	breast	27	breast	445	205	0.46
	c.4612C > T; p.Gln1538^a^	stop gained	M2002389	Luminal B (HER2+)	breast	38	breast	634	311	0.49
	c.4987–2A > G	splice site	M1814354	Luminal B (HER2-)	breast	43	breast	6,154	2,769	0.45
	c.5074G > A; p.Asp1692Asn	splice site	M2003980	Luminal B (HER2-)	breast	65	breast; ovary	597	287	0.48
	c.5089T > C; p.Cys1697Arg	missense	M1908879	HER2+	breast	36	breast	622	286	0.46
	c.5239C > T; p.Gln1747^a^	stop gained	M1822270	TNBC	breast	28	breast	3,201	1,568	0.49
	c.5251C > T; p.Arg1751^a^	stop gained	M1919481	Luminal B (HER2-)	breast	40	—	629	308	0.49
	c.5357T > C; p.Leu1786Pro	missense	M2001475	HER2+	breast	32	ovary	644	277	0.43^a^
	c.5470_5477del; p.Ile 1824fs	frameshift	M1903211	TNBC	breast	28	—	621	273	0.44^a^
	—	—	M1915850	Luminal B (HER2+)	breast	29	breast	2,226	868	0.39^a^
	—	—	M1913849	HER2+	breast	31	—	608	268	0.44^a^
	—	—	M2004099	HER2+	breast	31	—	661	304	0.46
	—	—	D2000656	TNBC	breast	40	breast	665	299	0.45
	—	—	M2001617	TNBC	breast	46	ovary	642	289	0.45
	—	—	M1916970	TNBC	breast	50	breast	623	287	0.46
	—	—	M1913669	TNBC	breast	30	-	608	292	0.48
	c.5503C > T; p.Arg 1835^a^	stop gained	M1921070	TNBC	breast	30	breast; ovary	634	311	0.49
	c.66dup; p.Glu23Argfs	frameshift	M1903763	TNBC	breast	29	breast	654	301	0.46
	c.869del; p.Leu290fs	frameshift	M1912969	TNBC	breast	41	breast	613	294	0.48
	c.981_982del; p.Cys328fs	frameshift	M2001103	TNBC	breast	37	-	468	225	0.48
	—	—	M1914021	TNBC	bilateral breast	46	-	499	235	0.47
	exon8del	large genomic rearrangement	N1861013	Luminal B (HER2+)	breast	29	breast	-	-	0.99
*BRCA2*	c.1238del; p.Leu413fs	frameshift	M2001692	Luminal B (HER2+)	breast	35	breast	425	196	0.46
	c.3109C > T; p.Gln1037^a^	stop gained	M1916464	Luminal B (HER2+)	breast	30	breast	316	158	0.50
	c.-39–1_-39del	splice site	M1908841	Luminal B (HER2+)	breast	26	—	534	246	0.46
	—	—	M1922853	TNBC	breast	47	—	529	233	0.44^a^
	c.4581del; p.Ser1528fs	frameshift	M2002400	Luminal B (HER2+)	breast	71	ovary	442	208	0.47
	c.5189del; p.Asn1730fs	frameshift	M2004286	Luminal B (HER2+)	breast	37	—	428	227	0.53
	c.5645C > A; p.Ser 1882^a^	stop gained	M1911239	Luminal B (HER2+)	breast	29	breast	225	110	0.49
	c.582G > A; p.Trp194^a^	stop gained	M1811133	Luminal B (HER2+)	breast	47	breast	3,325	1,596	0.48
	c.7988_8000delinsCA; p.Glu2663fs	frameshift	M2003796	Luminal B (HER2+)	breast	44	breast	438	193	0.44^a^
	c.8479_8485del; p.Pro2827fs	frameshift	N1845679	Luminal B (HER2+)	breast	31	breast	380	175	0.46
	c.8987del; p.Leu2996fs	frameshift	M2002180	Luminal B (HER2+)	bilateral breast	33	-	569	307	0.54
*BRIP1*	c.2464dup; p.Tyr822fs	frameshift	M1912924	Luminal B (HER2+)	breast	40	—	619	322	0.52
*CHEK2*	c.161_164del; p.His54fs	frameshift	N1853565	Luminal B (HER2-)	breast	27	breast	439	211	0.48
	c.432dup; p.Arg145fs	frameshift	M1914089	Luminal B (HER2+)	breast	27	breast	470	230	0.49
*ERCC3*	c.1854_1867del; p.Glu619fs	frameshift	M1911225	TNBC	breast	43	—	640	288	0.45
*FANCA*	c.2733G > A; p.Trp911^a^	stop gained	M1911710	TNBC	breast	41	—	561	292	0.52
	c.2923_2924del; p.Gly975fs	frameshift	M2002746	Luminal B (HER2+)	breast	32	breast	682	334	0.49
*HOXB13*	c.179del; p.Pro60fs	frameshift	M2002924	TNBC	breast	37	—	606	303	0.50
*MLH1*	c.1038G > C; p.Gln346His	splice site	M1921070	TNBC	breast	30	breast; ovary	557	256	0.46
*MRE11*	c.1897C > T; p.Arg633^a^	stop gained	M2001784	HER2+	breast	32	breast	671	302	0.45
*PALB2*	c.1784del; Asp595Valfs	frameshift	M1812984	TNBC	breast	42	breast	3,469	1,561	0.45
	c.3114-1G > A	splice site	M1916465	Luminal B (HER2+)	breast	46	breast	578	272	0.47
	c.3256C > T; p.Arg1086^a^	stop gained	N1912271	Luminal B (HER2+)	breast	32	breast	442	230	0.52
	c.3507_3508del; p.His1170fs	frameshift	N1730221	Luminal B (HER2+)	breast	29	breast	532	239	0.45
	c.751C > T; p.Gln251^a^	stop gained	M1923379	Luminal B (HER2+)	breast	45	breast	375	176	0.47
*PMS2*	exon14-15cn_del	large genomic rearrangement	M1907635	Luminal B (HER2+)	breast	33	—	—	—	0.46
*RAD51C*	c.1000G > T; p.Glu334^a^	stop gained	M2002180	Luminal B (HER2+)	bilateral breast	33	—	641	301	0.47
*RAD51D*	c.270_271dup; p.Lys91fs	frameshift	N1729354	HER2+	breast	30	breast	439	220	0.50
	—	—	M1916030	HER2+	breast	32	—	552	270	0.49
	c.556C > T; p.Arg186^a^	stop gained	M2001617	TNBC	breast	46	ovary	596	286	0.48
*RAD54L*	c.1403_1404del; p.Val468fs	frameshift	M1920663	Luminal B (HER2+)	breast	37	—	517	227	0.44^a^
*TP53*	c.328dup; p.Arg110fs	frameshift	M2001784	HER2+	breast	32	breast	394	197	0.50
	c.637C > T; p.Arg213^a^	stop gained	M2002098	Luminal B (HER2+)	breast	43	breast	514	221	0.43^a^
	c.733G > A; p.Gly245Ser	missense	N1831892	Luminal B (HER2-)	breast	27	breast	379	193	0.51

a Variants (single nucleotide variants and insertion-deletions) whose allele frequency were lower than 45% have been validated with Sanger sequencing.

### Variants of Uncertain Significance

Apart from pathogenic or likely pathogenic variants, 312 unique variants of uncertain significance (VUS) were identified among 175 (76.1%, 175/230) patients and 65 different genes. Of those, the most frequent genes were *RAD54L* (5.4%), followed by *ATM* (5.1%) and *FANCA* (4.6%). Table 6 summarizes all the VUSs identified in high penetrance breast cancer predisposition genes (*BRCA1*, *BRCA2*, *CDH1*, *PALB2*, *PTEN* and *TP53*).

**TABLE 6:** List of VUSs in high penetrance breast cancer predisposition genes.

Gene	Mutation variant	Mutation Type	Allele Frequency	SIFT	PolyPhen-2
*BRCA1*	c.134+4dup	splice site	—	—	—
	c.2481A > C; p.Glu827Asp	missense	0.000004	0.09	0.032
	c.3034A > G; p.Arg1012Gly	missense	—	0.14	0.442
	c.3172A > G; p.Ile1058Val	missense	—	0.7	0.008
	c.3596C > T; p.Ala1199Val	missense	0.000071	0.05	0.427
	c.3649T > C; p.Ser1217Pro	missense	0.000085	0.07	0.073
	c.4484+5G > A	intron	—	—	—
	c.5096G > A; p.Arg1699Gln	missense	0.00002	0	0.999
	c.5380G > A; p.Glu1794Lys	missense	—	0.14	0.259
*BRCA2*	c.2946A > G; p.Ile982Met	missense	0.000088	0.08	0.019
	c.4376A > G; p.Asn1459Ser	missense	0.000043	0.82	0.172
	c.4436G > A; p.Ser1479Asn	missense	—	0.57	0.006
	c.5191C > T; p.His1731Tyr	missense	0.0000082	0.24	0.94
	c.6131G > T; p.Gly2044Val	missense	0.00002	0.02	0.079
	c.7088A > G; p.Tyr2363Cys	missense	0.000004	0.12	0.014
	c.7601C > T; p.Ala2534Val	missense	0.000096	0.67	0.499
	c.7979_7984del; p.Tyr2660_Thr2662delinsSer	in frame del	—	—	—
	c.8356G > A; p.Ala2786Thr	missense	0.000057	0.03	1
	c.8518A > G; p.Ile2840Val	missense	0.000011	1	0.077
	c.8682A > C; p.Gln2894His	missense	—	0.02	1
	c.8702G > A; p.Gly2901Asp	missense	0.00013	0	1
	c.9665G > A; p.Cys3222Tyr	missense	—	0.16	0.039
*PALB2*	c.1213C > G; p.Pro405Ala	missense	0.000025	0	1
	c.1492G > T; p.Asp498Tyr	missense	0.00049	0.12	0.904
	c.1556C > A; p.Ala519Asp	missense	0.000008	0.23	0.418
	c.1659C > A; p.His553Gln	missense	0.000024	0.19	0.002
	c.2509G > A; p.Glu837Lys	missense	0.000099	0.25	0.922
	c.2586 + 18T > A	intron	—	—	—
	c.308G > A; p.Gly103Glu	missense	—	1	0
	c.3379T > C; p.Cys1127Arg	missense	0.000008	0.35	0.761
	c.5A > T; p.Asp2Val	missense	—	0.07	0.018
*TP53*	c.145G > A; p.Asp49Asn	missense	0.000008	0.06	0.358
	c.776A > T; p.Asp259Val	missense	—	0.07	0.537
	c.91G > A; p.Val31Ile	missense	0.00023	0.84	0.001

## Discussion

The prevalence and spectrum of germline mutations in breast cancer patients vary across ethnicities (Hall et al., 2009; Han et al., 2011; Kwong et al., 2016). Compared with women of Western European descent (12.1%), women in Africa (15.6%) and Latin America (14.8%) had a higher incidence of *BRCA1/2* pathogenic mutations (Hall et al., 2009). Data for the Asian population showed that the prevalence of *BRCA1/2* germline mutations was 9.8% in South Korean non-familial high-risk breast cancer patients (Han et al., 2011) and 9.4% in Chinese hereditary breast-ovarian cancer (HBOC) families (Kwong et al., 2016). The application of NGS has enabled sequencing a large number of genes simultaneously, and thus, many other non-*BRCA* breast cancer susceptibility genes have been identified. The NCCN guideline list the susceptibility genes of hereditary breast cancer, including 6 high penetrance (*BRCA1*, *BRCA2*, *CDH1*, *PALB2*, *PTEN* and *TP53*) and 9 moderate to low penetrance (*ATM*, *BARD1*, *BRIP1*, *CHEK2*, *NBN*, *NF1*, *RAD51C*, *RAD51D* and *STK11*) susceptibility genes, most of which also participate in HRR pathway (Nielsen et al., 2016).

However, only a few studies to date report the prevalence of non-*BRCA* genes in the Chinese population. Samuel Guan Wei Ow et al. analyzed 419 Asian patients suspected to have hereditary breast cancer syndrome who underwent genetic testing and found that the frequency of detrimental mutations in non-*BRCA* genes varied from 0 to 13.3% due to differences in ethnicity (Ow et al., 2019). Using a 40 gene panel, another study found a non-*BRCA* gene mutation rate of 6.8% in a cohort of 937 Chinese breast cancer patients, with *TP53* (1.9%), *PALB2* (1.2%), *CHEK2* (0.6%) and *ATM* (0.6%) being the major non-*BRCA* genes identified (Li et al., 2019). A recent study analyzed 1,338 Chinese high-risk breast cancer patients who tested mutation negative by a four-gene panel (*BRCA1*, *BRCA2*, *PTEN* and *TP53*) and found that pathogenic variants in cancer predisposition genes beyond *BRCA1*, *BRCA2*, *PTEN* and *TP53* were detected in an additional 4.6% of patients using a multigene panel, with *PALB2* (1.4%), *RAD51D* (0.8%) and *ATM* (0.8%) being the most commonly mutated genes (Kwong et al., 2020). Herein, we retrospectively analyze 230 high-risk breast cancer patients who had undergone peripheral blood germline 72 multigene panel testing, with a *BRCA* mutation rate of 19.1%; 12.2% of the patients carried non-*BRCA* mutations. Consistent with previous studies, the major mutated non-*BRCA* genes were *PALB2* (2.2%), *ATM* (1.7%), *RAD51D* (1.3%) and *TP53* (1.3%).

Apart from the high-frequency mutated genes, pathogenic variants were also detected in another 11 non-*BRCA* genes (*ATR*, *BARD1*, *BRIP1*, *ERCC3*, *FANCA*, *HOXB13*, *MLH1*, *MRE11*, *RAD51C*, *RAD54L* and *PMS2*). In addition to the hereditary breast cancer susceptibility genes listed by NCCN, *MLH1* and *PMS2* are risk genes recommended by NCCN for multigene testing. However, there is insufficient evidence to prove that *MLH1* and *PMS2* are related to breast cancer risk and mutation carriers need to be managed based on family history. The remaining 6 genes, *ATR*, *ERCC3*, *FANCA*, *HOXB13*, *MRE11* and *RAD54L*, are not included in the risk genes recommended by NCCN guidelines. Among them, *ATR*, *FANCA*, *MRE11* and *RAD54L* are HRR genes. *MRE11*, together with *RAD50* and *NBS1* (*MRE11-RAD50-NBS1* complex), locates to the end of the double-strand breaks (DSBs) locus (Kinner et al., 2008; Wang et al., 2014) and activates *ATM* through the interaction between *ATM* and *NBS1* (Bian et al., 2019), triggering ataxia telangiectasia and *ATR* activation. *ATM* and *ATR* then phosphorylate downstream targets, including *BRCA1* and *CHEK2*. *RAD54L* binds double-strand DNA and induces a DNA topological change, which is thought to facilitate homologous DNA paring and stimulate DNA recombination. *FANCA* participates in the Fanconi anemia (FA) pathway, which is involved in DNA interstrand cross-link (ICL) damage repair and crucial for maintaining the integrity of the genome (Nakanishi et al., 2011). *ERCC3* encodes an ATP-dependent DNA helicase that plays a role in the DNA nucleotide excision repair (NER) pathway. *HOXB13* encodes a transcription factor that belongs to the homeobox gene family and plays a role in fetal skin development and cutaneous regeneration. *HOXB13* gene mutations are often detected in prostate cancer patients. The correlation between these genes and the risk of hereditary breast cancer is not clear yet, and further studies are needed.

9.6% of high-risk breast cancer patients in our study carried non-*BRCA* HRR gene mutations. The current understanding of specific HRR genes is not comprehensive. Dana Sherill-Rofe and colleagues followed the coevolution of the HRR pathway across the eukaryotic life tree and defined a gold standard list of 79 well-established HRR genes. According to their function, these genes are divided into 6 parts: DSB recognition, end resection, FA pathway, regulation (DNA damage response), strand invasion and D-loop formation, synthesis and holiday junction processing (Pilié et al., 2019). Referring to the results of Dana Sherill-Rofe and taking intersection with the 72 multigene panel used in our study, we obtained 18 genes and defined these genes as HRR genes, including *ATM*, *ATR*, *BARD1*, *BRCA1*, *BRCA2*, *BRIP1*, *CHEK1*, *CHEK2*, *FANCA*, *FANCI*, *MRE11A*, *NBN*, *PALB2*, *RAD50*, *RAD51B*, *RAD51C*, *RAD51D* and *RAD54L*. Most patients with germline HRR gene mutations are not sporadic and have at least one first and/or second-degree relatives who also have a tumor, which indicates that the germline mutations of HRR genes are closely related to heredity (Li et al., 2008). In addition, mutations of non-*BRCA* HRR genes show similar PARPi sensitivity as *BRCA1/2*. PARPi can lead to cell death via a synergistic effect known as “synthetic lethality” with homologous recombination deficiency (HRD) (Zhang et al., 2012). The presence of pathogenic mutations in different non-*BRCA* HRR genes leads to difference responses to PARPi therapy. Abida et al. studied the response to the PARPi rucaparib in metastatic castration-resistant prostate cancer (mCRPC) patients with non-*BRCA* DNA damage repair pathogenic gene mutations and found that tumors with *ATM*, *CDK12* and *CHEK2* mutations had a limited response to rucaparib, while tumors with *BRIP1*, *FANCA*, *PALB2* and *RAD51B* mutations benefitted from PARPis (Lin et al., 2016). In addition to PARP, *ATM*, *ATR* and *CHEK1/2* may also be used for targeted therapy. The first *ATR* inhibitor, M6620, has been tested, and *ATM* inhibitors (such as M3541) are in clinical trials (Zhang et al., 2016; Lang et al., 2017).

In terms of each risk factor, our results suggest that the frequencies of *BRCA* mutations in early-onset breast cancer, familial breast cancer, TNBC and bilateral breast cancer (19.4–38.5%) are higher than those observed in other Chinese population studies (2.3–12.5%) (Gavande et al., 2016; Li et al., 2019; Sherill-Rofe et al., 2019; Abida et al., 2020; Hirsch et al., 2020; Foo et al., 2021) and the frequencies of non-*BRCA* mutations are also at a high level (7.7–17.5%). However, no pathologic variants were observed in the male patients in our study. In contrast, previous studies reported a high *BRCA1/2* mutation frequency of 15.2–15.4% and a non-*BRCA* mutation rate of 15.4% in Chinese male breast cancer patients (Gavande et al., 2016; Li et al., 2019). Among high-risk factors, family history showed a correlation with both *BRCA* (*p* = 0.005) and non-*BRCA* HRR gene mutation status (*p* = 0.036). Besides, TNBC showed a correlation with *BRCA1* gene mutation status (*p* = 0.038).

Almost all high-risk patients in our study met the standards of *the expert consensus on the recommendation of BRCA genetic testing for breast cancer patients in China* (Wang et al., 2018). The application of a multigene panel helped us to find 28 non-BRCA gene mutation carriers among 230 high-risk breast cancer patients. The latest *consensus guidelines on genetic testing for hereditary breast cancer* by the American Society of Breast Surgeons (ASBrS) recommends that all breast cancer patients should undergo multigene testing to assess whether they are at risk of hereditary cancer (Manahan et al., 2019). *The Chinese Anti Cancer Association (CACA) breast cancer diagnosis and treatment guidelines (2019)* recommends multigene testing for the following three situations: 1) patients with a personal or family history of tumor; 2) testing results can be fully explained; 3) testing results are helpful to clinical screening, diagnosis and treatment (Breast Cancer Professiona, 2019). However, in current clinical practice, multigene testing is mainly used in advanced breast cancer patients who have failed by multiline therapies in China. A recent study conducted multigene panel testing for breast cancer patients who had not undergone genetic testing and found that up to 50% of carriers of pathogenic or likely pathogenic mutations did not meet the NCCN guidelines (O’Leary et al., 2017; Beitsch et al., 2019; González-Santiago et al., 2020). All these indicate that the current standards for tumor polygene detection are still not perfect.

Among our cohort, 312 unique VUSs were identified among 175 (76.1%) patients and 65 different genes. However, the VUSs found in our cohort, including some rare variants, does not necessarily lead to the occurrence and development of tumors. The causes of tumors in these high-risk patients need to be further explored. On the one hand, reliable functional experiments are needed to reclassify the pathogenicity of VUSs. On the other hand, expanding genetic testing, such as whole-exome sequencing (WES) and whole genome sequencing (WGS), might help to reveal new breast cancer susceptibility genes.

Our research has some innovations and limitations. We focused on the prevalence and spectrum of non-*BRCA*, especially non-*BRCA* HRR gene germline mutations in the Chinese population, which is very rare in previous studies. Meanwhile, we analyzed the correlations between the high-risk factors and non-*BRCA* and non-*BRCA* HRR gene mutation status, which showed consistency with previous studies. However, as a retrospective study, the sample size for each risk factor was not controlled for, especially for the male and bilateral breast cancer groups, which limited the discovery of other possible variations.

In summary, 12.2% of high-risk breast cancer patients in our study carried non-*BRCA* gene mutations, with *PALB2* (2.2%), *ATM* (1.7%), *RAD51D* (1.3%) and *TP53* (1.3%) being the major non-*BRCA* genes mutated. As a *BRCA*-related family history is associated with HRR mutations, we strongly suggest that breast cancer patients with a *BRCA*-related family history receive comprehensive gene mutation testing, especially HRR genes, which are not only related to high risk of breast cancer, but also potentially related to PARPi targeted therapy in China. The exact relationship of rare gene mutations to breast cancer predisposition and the pathogenicity of VUSs need to be further investigated. With the widespread use of NGS technology, we are expecting to discover more breast cancer susceptibility genes. Guidelines for multigene testing and management of mutation carriers should be compiled to benefit Chinese breast cancer patients.

## Data Availability

The original contributions presented in the study are publicly available. This data can be found here: https://www.biosino.org/node/project/detail/OEP002708
